# Circulating metabolite signatures indicate differential gut-liver crosstalk in lean and obese MASLD

**DOI:** 10.1172/jci.insight.180943

**Published:** 2025-03-18

**Authors:** Mathias Haag, Stefan Winter, Aurino M. Kemas, Julia Tevini, Alexandra Feldman, Sebastian K. Eder, Thomas K. Felder, Christian Datz, Bernhard Paulweber, Gerhard Liebisch, Oliver Burk, Volker M. Lauschke, Elmar Aigner, Matthias Schwab

**Affiliations:** 1 Dr. Margarete Fischer-Bosch Institute of Clinical Pharmacology, Stuttgart, Germany.; 2 University of Tübingen, Tübingen, Germany.; 3 Department of Physiology and Pharmacology, Karolinska Institutet, Stockholm, Sweden.; 4 Department of Laboratory Medicine, Paracelsus Medical University, Salzburg, Austria.; 5 Obesity Research Unit, Paracelsus Medical University, Salzburg, Austria.; 6 Department of Internal Medicine I, Paracelsus Medical University, Salzburg, Austria.; 7 Department of Internal Medicine, Hospital Oberndorf, Oberndorf, Austria.; 8 Institute of Clinical Chemistry and Laboratory Medicine, University Hospital Regensburg, Regensburg, Germany.; 9 Center for Molecular Medicine, Karolinska Institutet and University Hospital, Stockholm, Sweden.; 10 Departments of Clinical Pharmacology and of Biochemistry and Pharmacy, University Hospital Tübingen, Tübingen, Germany.

**Keywords:** Hepatology, Metabolism, Obesity

## Abstract

**BACKGROUND:**

Alterations in circulating metabolites have been described in obese metabolic dysfunction–associated steatotic liver disease (MASLD), but data on lean MASLD are lacking. We investigated serum metabolites, including microbial bile acids and short-chain fatty acids (SCFAs), and their association with lean and obese MASLD.

**METHODS:**

Serum samples from 204 people of European descent were allocated to four groups: lean healthy, lean MASLD, obese healthy, and obese MASLD. Liquid chromatography–mass spectrometry–based metabolomics and linear model analysis were performed. MASLD prediction was assessed based on least absolute shrinkage and selection operator regression. Functional effects of altered molecules were verified in organotypic 3D primary human liver cultures.

**RESULTS:**

Lean MASLD was characterized by elevated isobutyrate, methionine sulfoxide, propionate, and phosphatidylcholines. Patients with obese MASLD had increased sarcosine and decreased lysine and asymmetric dimethylarginine. Using metabolites, sex, and BMI, MASLD versus healthy could be predicted with a median AUC of 86.5% and 85.6% in the lean and obese subgroups, respectively. Functional experiments in organotypic 3D primary human liver cultures showed propionate and isobutyrate induced lipid accumulation and altered expression of genes involved in lipid and glucose metabolism.

**CONCLUSION:**

Lean MASLD is characterized by a distinct metabolite pattern related to amino acid metabolism, lipids, and SCFAs, while metabolic pathways of lipid accumulation are differentially activated by microbial metabolites. We highlight an important role of microbial metabolites in MASLD, with implications for predictive and mechanistic assessment of liver disease across weight categories.

**FUNDING:**

Robert Bosch Stiftung, Swedish Research Council (2021-02801, 2023-03015, 2024-03401), ERC Consolidator Grant 3DMASH (101170408), Ruth and Richard Julin Foundation for Gastroenterology (2021-00158), SciLifeLab and Wallenberg National Program for Data-Driven Life Science (WASPDDLS22:006), Novo Nordisk Foundation (NNF23OC0085944, NNF23OC0084420), PMU-FFF (E-18/28/148-FEL).

## Abstract 2

Our study provides evidence that there are differences in circulating microbial short-chain fatty acids between lean patients with Metabolic Dysfunction-associated Steatotic Liver Disease (MASLD) compared to obese patients.

## Graphical abstract

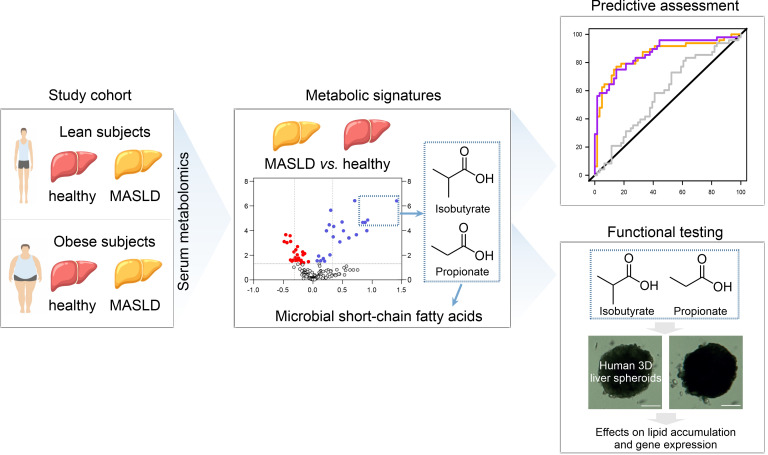

## Introduction

Metabolic dysfunction–associated steatotic liver disease (MASLD) is the most common chronic liver condition, affecting approximately 30% of the population in Western countries. The histological spectrum of MASLD ranges from simple steatosis to metabolic dysfunction–associated steatohepatitis (MASH) and liver fibrosis. Despite vast efforts, a comprehensive understanding of the pathogenesis of MASLD, which involves complex interactions between environmental factors, obesity, changes in the microbiota, and predisposing genetic variants, remains elusive (1–4). While MASLD is mostly associated with obesity, MASLD is also becoming increasingly prevalent in individuals with normal weight, with an estimated global prevalence of 4.1%, most commonly in Asia (5). Lean MASLD is thereby recognized as mechanistically different from obese MASLD and is characterized by distinct clinical and metabolic alterations (6, 7), severe insulin resistance (8), and an increased risk of liver-related mortality (9).

As steatotic liver is commonly associated with metabolic dysfunction (10, 11), metabolomic analyses have become valuable tools for identifying metabolic signatures that are characteristic of MASLD (12, 13). These analyses facilitate hypothesis generation and, when validated by in vitro assays, enable functional investigation of the observed effects. This approach thereby provides mechanistic insights into the underlying mechanisms of the disease. While metabolomics has allowed differentiation of MASLD subtypes (14), investigations of metabolic signatures that differ between lean and obese MASLD are limited (7).

Notably, a recent study indicated qualitative differences in the composition of the intestinal microbiome between lean and obese MASLD groups (15), thus pointing to different pathways along the gut-liver axis and a potential involvement of microbial metabolites (16).

Secondary bile acids (BAs) and short-chain fatty acids (SCFAs) are two important microbial metabolite classes (17–19) that rapidly reach the liver because of efficient intestinal absorption, where they can influence hepatic lipid and glucose metabolism (20) as well as inflammatory signaling (21). For BAs, this is mediated via activation of cell surface (TGR5) and nuclear receptors (FXR, NR1H4). Likewise, SCFAs directly link the gut microbiome to hepatic metabolism through G protein–coupled receptor (GPCR) signaling via GPR41, GPR43, and GPR109a. Therefore, targeting BA-related pathways, e.g., through modification of BA transporters, nuclear receptor modulation, or SCFA-sensing GPCRs has emerged as a potential therapeutic strategy for MASLD treatment (22, 23). Although BAs are well described for their function as regulatory molecules, SCFAs also play important roles as substrates for hepatic glucose, carbohydrate, and lipid metabolism (24).

In this study, we aim to provide a mechanistic explanation for the occurrence of lean and obese MASLD under consideration of metabolomics and lipidomics. Since we tried to capture the microbiome, special emphasis was placed on the evaluation of SCFAs and BAs as microbial metabolites. Consistent with this aim, we used organotypic 3D liver spheroids to allow for functional attribution of the observed effects. In addition, we describe metabolic signatures to discriminate between lean/obese MASLD and healthy participants based on comprehensive serum metabolomics.

## Results

### Clinical data of lean and obese MASLD and BMI-matched healthy controls.

**Figure 1:**
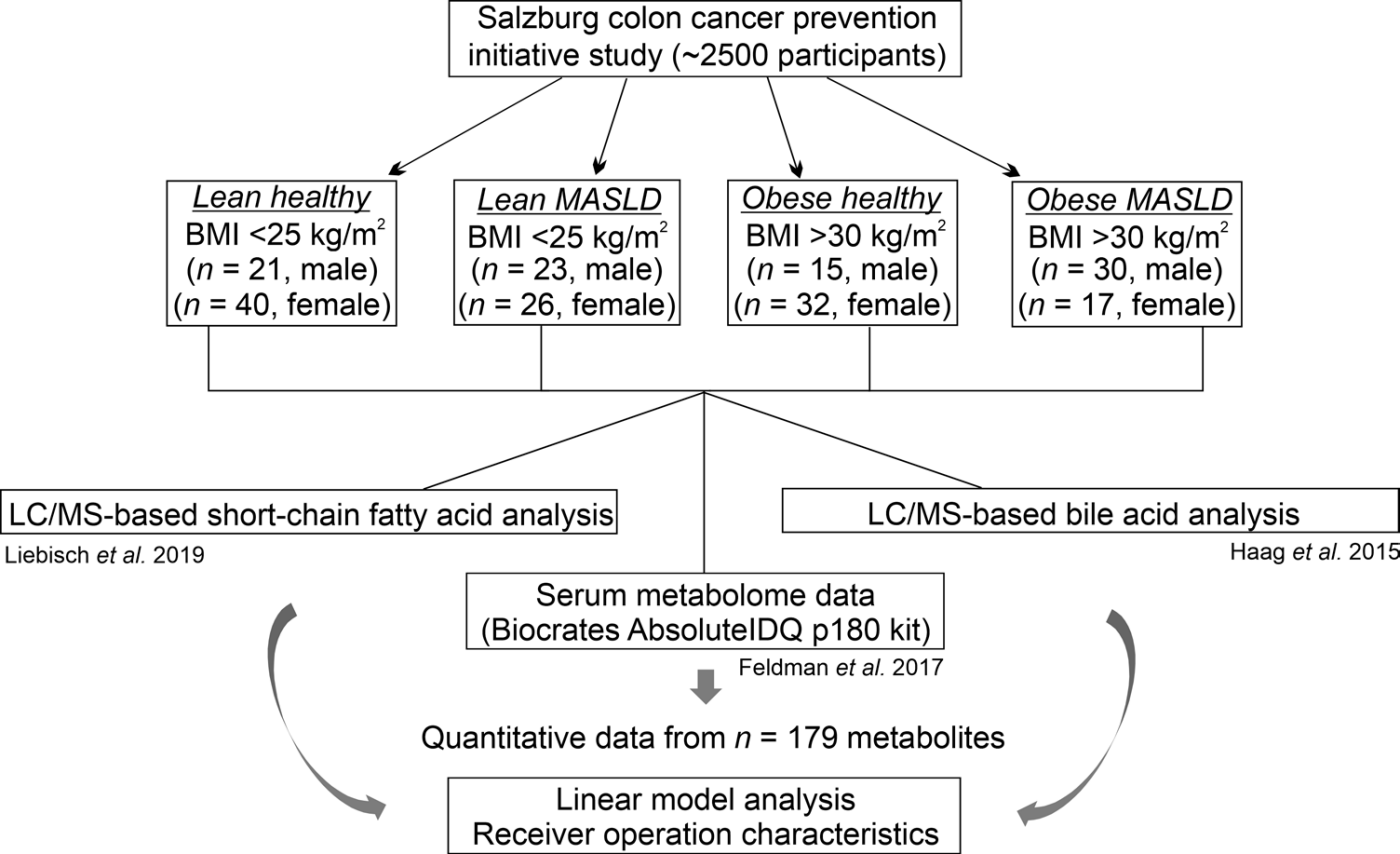
Overview of study cohorts, metabolomics measurements, and statistical analysis. Lean and obese patients with MASLD and BMI-matched healthy controls were allocated from the Salzburg Colon Cancer Prevention Initiative study (SAKKOPI) to the indicated patient groups (6). The study design and details of the clinical and biochemical workup of included participants have been reported previously (85). MASLD was diagnosed based on ultrasound analysis. Quantitative data from BA and SCFA measurements were merged with previously acquired metabolomics data (6), and the resulting *n* = 179 metabolic traits were analyzed by linear model analysis in order to detect metabolites that differ between lean and obese MASLD and least absolute shrinkage and selection operator–based (LASSO-based) AUC receiver operating characteristic (ROC) analysis for MASLD versus healthy prediction.

A total of 204 participants of European descent with or without clinically established MASLD were analyzed (96 MASLD and 108 non-MASLD controls). Figure 1 shows an overview of the assigned lean and obese MASLD and corresponding healthy control groups as well as the metabolomics measurements and statistical analyses performed. With respect to clinical parameters, both lean and obese MASLD groups were characterized by significantly higher levels of liver enzymes compared with BMI-matched healthy controls (alanine aminotransferase, ALT; gamma-glutamyl transferase, GGT; adjusted *P* ≤ 0.01; Table 1). In addition, groups with MASLD presented features of the metabolic syndrome, such as an increased prevalence of diabetes and significantly elevated triglycerides (hypertriglyceridemia), fasting glucose, and fasting insulin, accompanied by reduced serum HDL-C (all adjusted *P* ≤ 0.05). MASLD fibrosis scores (FIB4 and NFS) were not significantly different between MASLD and BMI-matched control groups (adjusted *P* ≥ 0.2; indicating nonadvanced MASLD stages in the overall cohort). The antiinflammatory mediator adiponectin was significantly reduced in both lean and obese MASLD, while pro-inflammatory IL-6 was lower in lean and higher in obese MASLD compared with the BMI-matched healthy controls. Glycosylated hemoglobin (HbA1C), a marker of uncontrolled hyperglycemia and a potential risk factor for MASLD (25), was significantly elevated in people with obesity (adjusted *P* value < 0.01) but not in people with normal weight and MASLD (adjusted *P* value 0.4).

### Sex differences in metabolomic profiles support the integrity of the clinical cohort.

**Figure 2:**
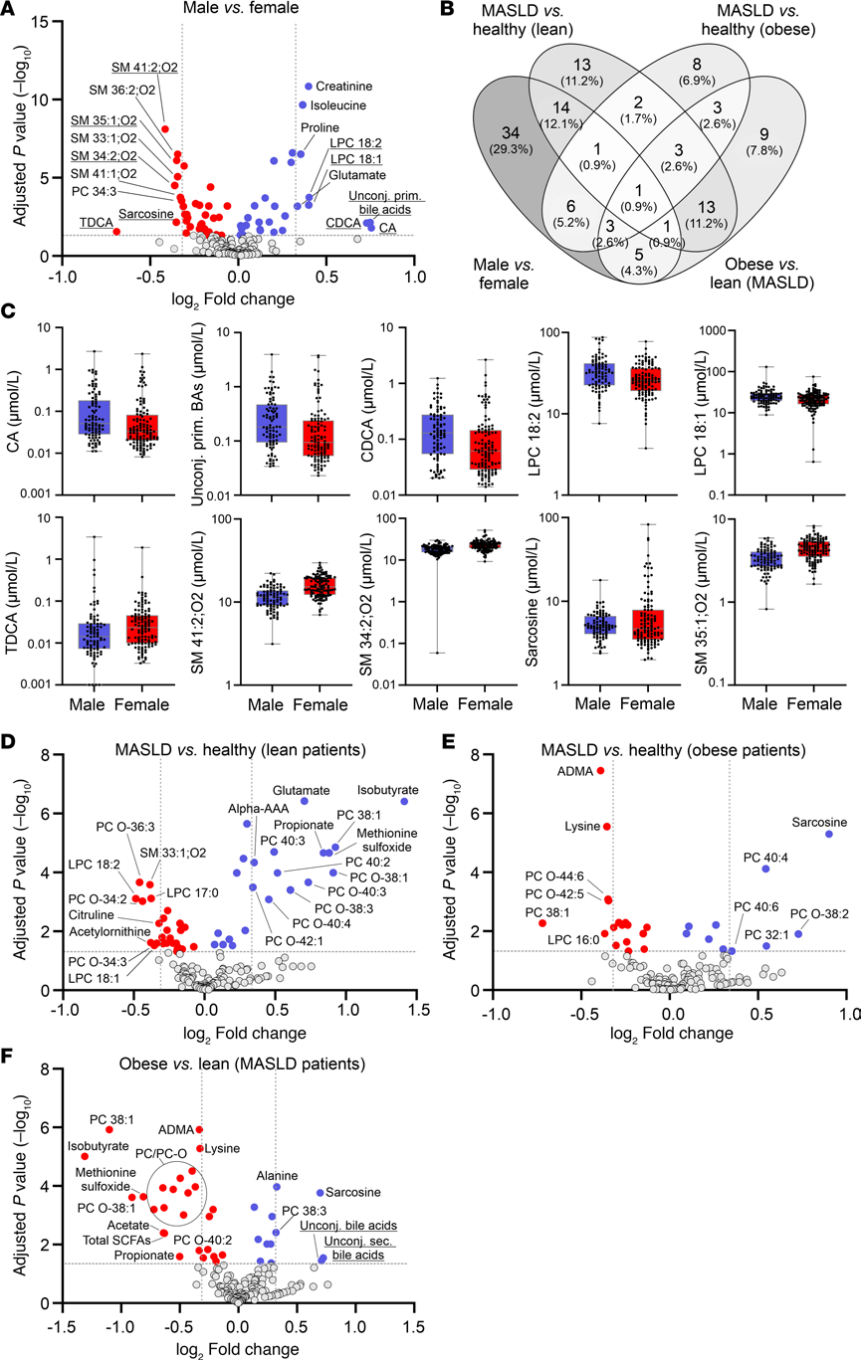
Serum metabolite profiles vary by sex and exhibit distinct metabolic signatures in lean and obese MASLD. (**A**) Volcano plot displaying metabolites found at significantly (adjusted *P* value < 0.05) higher (blue) or lower (red) levels in males compared with females. Statistical analyses were performed in the complete cohort (*n* = 204) irrespective of MASLD and BMI. Metabolites that exhibit a log_2_ fold-change > 0.3125 and an adjusted *P* value < 0.05 are labeled with the corresponding names. The top 10 significant metabolites with the highest log_2_ fold-changes are underlined and shown as box plots in **C** (see Supplemental Figure 2 for box plots of other significantly altered metabolites). The upper/lower borders of a box are defined by the first/third quartile while the line within a box represents the median. Whiskers extend to the highest or lowest values. (**B**) Venn diagram representing metabolites that are significantly (adjusted *P* values < 0.05, Supplemental Tables 2–5) changed for the indicated group comparisons. (**D**) Volcano plot displaying significantly (adjusted *P* value < 0.05) upregulated (blue) or downregulated (red) metabolites in lean MASLD compared with BMI-matched healthy controls (*n* = 110). (**E**) Volcano plot displaying significantly (adjusted *P* value < 0.05) upregulated (blue) or downregulated (red) metabolites in obese MASLD compared with BMI-matched healthy controls (*n* = 94). (**F**) Volcano plot displaying metabolites found at significantly (adjusted *P* value < 0.05) higher (blue) or lower (red) levels in obese compared with lean patients with MASLD (*n* = 96). Metabolites in **D**–**F** that exhibit a log_2_ fold-change > 0.3125 and an adjusted *P* value < 0.05 are labeled with the metabolite names (corresponding box plots are displayed in Supplemental Figures 3–7). Data were analyzed by linear model analysis for volcano plot generation.

Lack of standardization in the collection of clinical biosamples can have a major impact on the level of circulating metabolite profiles. We therefore analyzed sex differences in serum metabolomic profiles as a proxy to assess the integrity of the clinical cohort. Linear model analysis demonstrated a total of 65 metabolites that were significantly altered between men and women, irrespective of MASLD and BMI (Figure 2A and Supplemental Table 2; supplemental material available online with this article; https://doi.org/10.1172/jci.insight.180943DS1). Specifically, men had elevated amounts of unconjugated primary BAs (cholic acid [CA] and chenodeoxycholic acid [CDCA]) and the lyso-phosphatidylcholines (lyso-PCs) 18:2 and 18:1 (Figure 2, A and C, and Supplemental Table 2). In addition, creatinine, several amino acids (i.e., proline, glutamate, tryptophan, kynurenine, isoleucine, leucine, and valine), and the acylcarnitines (ACars) propionylcarnitine (C3) and decenoylcarnitine (C10:1) were found at significantly higher levels in men (Supplemental Table 2 and Supplemental Figure 2). In contrast, the levels of sphingomyelins (SM 41:1;O2, SM 35:1;O2, SM 34:2;O2), taurodeoxycholate (TDCA), sarcosine, and most of the phosphatidylcholine species (e.g., PC 34:3, PC O-36:2) were lower in men compared with women (Figure 2, A and C; Supplemental Table 2; and Supplemental Figure 2).

These findings thus verify previous reports of substantial sex differences in circulating lipid profiles, including PCs and lyso-PCs (26). Our analysis is furthermore in accordance with known dissimilarities in CDCA levels (27) and BA synthesis (28) and an increase in branched-chain amino acids in men, whereas the sphingolipid pathway was increasingly activated in women (29). Similarly, our results are in line with recently reported sex differences of sarcosine and ACars (30, 31). While higher sarcosine concentrations in postmenopausal women suggest a link between menopause and the choline oxidation pathway, elevated ACars in men are associated with fasting status, indicating sex-specific differences in the maintenance of energy homeostasis. In summary, the detection of significant metabolic alterations related to sex demonstrates that sex, as a well-known variable influencing blood metabolite levels, can be robustly captured in the clinical cohort. This finding further verifies the preserved integrity (e.g., with respect to preanalytical sample processing; ref. 32) of the collected serum samples.

### Lean and obese MASLD exhibit characteristic profiles of circulating metabolites.

Lean and obese MASLD share abnormalities in blood lipid profiles, particularly elevated non-HDL cholesterol and hypertriglyceridemia, whereas body composition, gut microbiota, and susceptibility to environmental influences are dissimilar between both disease phenotypes (33). To identify metabolic fingerprints characteristic of the different MASLD etiologies, metabolomic profiles of a total of 179 metabolites were generated. Overall, we found that 48 and 27 metabolites were significantly different between MASLD and healthy controls in lean and obese cohorts, respectively (Figure 2, B, D, and E; Supplemental Figures 3–5; and Supplemental Tables 3 and 4). Of these, only 7 metabolites were found to be altered in both groups with MASLD, whereas 41 and 20 metabolites were specifically affected in either lean or obese participants (Figure 2B and Supplemental Tables 3 and 4). Interestingly, even metabolites that were altered in both groups were predominantly changed in opposite directions. Among the top 5 metabolites significantly elevated in patients with lean MASLD, we identified a marked increase in the SCFAs isobutyrate (Ibu) and propionate (Pro) (Figure 2D and Supplemental Table 3), whereas levels of these metabolites were not significantly increased in patients with obesity and MASLD (Figure 2E and Supplemental Table 4). A similar result was evident for methionine sulfoxide, which was significantly increased in lean (*P* = 2.2 × 10^–5^) but not in obese MASLD (*P* = 0.35). On the contrary, the lipid species PC O-36:3, PC O-34:2, lyso-PC 18:2, lyso-PC 17:0, and SM 33:1;O2 were significantly reduced in lean MASLD (Figure 2D).

A positive association of sarcosine levels with steatosis (*P* = 5 × 10^–6^) as well as an anticorrelation with the basic amino acids lysine and asymmetric dimethylarginine (ADMA) was specific for obese MASLD (Figure 2E and Supplemental Table 4). Other characteristic changes within the obese MASLD cohort were found for various PC species (i.e., elevations of PC 40:6, PC 40:4, PC 32:1, PC O-38:2; reduced levels of PC 38:1, lyso-PC 16:0, PC O-44:6, PC O-42:5).

Moreover, alterations related to overweight in MASLD were studied by comparing metabolite profiles between people with normal weight and people with obesity (excluding healthy controls). Here, we observed reduced levels of lipids (i.e., PC/PC-O), SCFAs, ADMA, and methionine sulfoxide and higher levels of sarcosine, alanine, and unconjugated BAs in obese MASLD compared with lean MASLD (Figure 2F, Supplemental Figures 6 and 7, and Supplemental Table 5).

**Figure 3:**
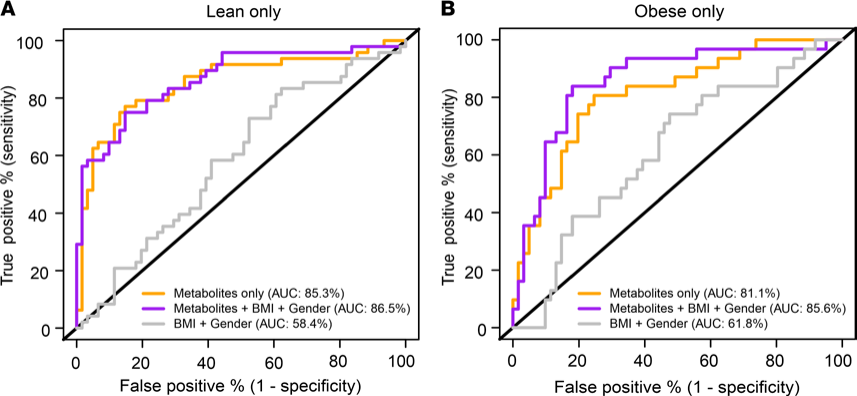
Assessment of the potential of serum metabolites for steatosis prediction. MASLD versus healthy prediction was assessed in (**A**) lean (*n* = 110) and (**B**) obese (*n* = 94) subgroups considering metabolites only (yellow curve), sex + BMI (gray curve), or sex + BMI + metabolites (magenta curve). Displayed ROC curves correspond to respective models with median AUC values (within 100 LASSO repeats). Variable selection and regularization was applied using LASSO regression.

To assess the potential of circulating metabolites for steatosis prediction, variable selection and regularization using least absolute shrinkage and selection operator (LASSO) regression was applied for classification based on all 179 metabolites, with and without additional consideration of BMI and sex. In the lean and obese subgroups, models based on metabolites alone showed median AUCs of 85.3% and 81.1% in 100 times repeated nested cross-validation, respectively, and only marginal improvements (median AUCs of 86.5% and 85.6%) when BMI and sex were also considered (Figure 3, A and B, and Supplemental Table 6). In contrast, models based on BMI and sex alone only achieved median AUCs of 58.4% and 61.8% in the lean and obese subgroups, respectively (Figure 3, A and B, and Supplemental Table 6), demonstrating that circulating metabolite profiles exhibited a higher predictive potential for MASLD prediction than physiological parameters.

Interestingly, when applying the metabolite-based models (with or without BMI and sex covariates) established in the subgroups with normal weight to the subgroups with obesity or vice versa, the AUCs were reduced by more than 30% (Supplemental Figure 8). Consistent with this observation, metabolite-based MASLD prediction in all patients (*n* = 204) resulted in median AUCs that were about 7%–10% lower compared with their group-specific performances (data not shown). Together, these results demonstrate that MASLD is associated with circulating metabolite profiles, which show pronounced differences between lean and obese MASLD, thus encouraging the assessment of MASLD-associated metabolites stratified by patient BMI.

### Pro and Ibu induce lipid accumulation by modulating gene expression in metabolic pathways.

Microbiota-associated SCFAs play a key role as regulators in MASLD (34), and the altered flux of these metabolites along the gut/liver axis may contribute to hepatic steatosis. To test this hypothesis, we treated organotypic primary human liver spheroids with Pro and Ibu, 2 microbial metabolites that were significantly associated with lean MASLD (Figure 2D).

**Figure 4:**
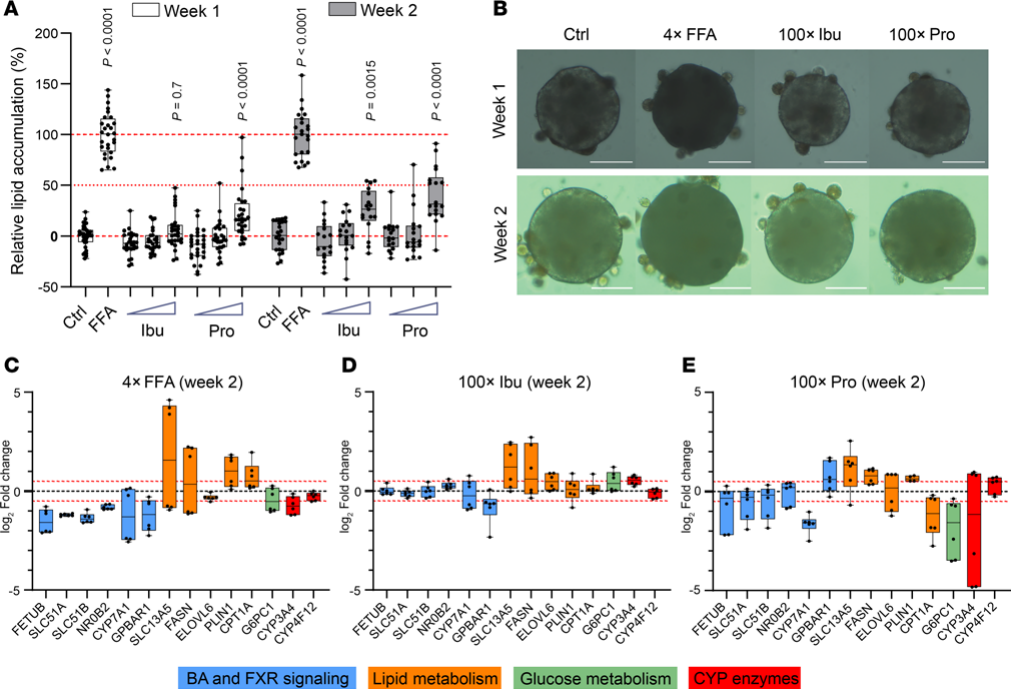
Pro and Ibu induce lipid accumulation in human 3D liver spheroids by altering gene expression in metabolic pathways. (**A**) Spheroids (*n* = 3 donors, with 8–10 individual spheroids per donor) were treated with FFAs or increasing concentrations (i.e., 1×, 10×, and 100× of quantified serum level) of Ibu and Pro for 2 weeks. Triglyceride levels were quantified weeks 1 and 2 and normalized to the control and FFA groups, respectively. Data are presented as box-and-whisker plots with points. Each dot represents an individual spheroid. Statistical analysis was performed by 1-way ANOVA with post hoc Dunnett’s multiple-testing correction against control (Ctrl) of respective week. *P* values are shown on top of box plots for statistically significant lipid accumulation against Ctrl. (**B**) Representative bright-field images of the spheroids from the experiment shown in **A**. Scale bars = 100 μm. (**C**) FFAs, (**D**) Ibu, and (**E**) Pro alter the expression of genes involved in lipid and glucose metabolism, BA/farnesoid X receptor (FXR) signaling, and cytochrome P450 (CYP) enzymes. Data are presented as box-and-whisker plots representing log_2_FC of gene expression compared with controls (*n* = 2 donors, each analyzed in triplicate). The red dotted line denotes a log_2_FC threshold of ± 0.5.

In addition to treatment with SCFAs, the spheroids were exposed to free fatty acids (FFAs), which served as a positive control to assess key molecules that are known to contribute to liver fat accumulation. As expected (35), FFAs induced a strong accumulation of intracellular triglycerides (Figure 4, A and B), coupled with a consistent upregulation (log_2_FC > 0.5) of a subset of genes involved in lipid metabolism, including perilipin 1 (*PLIN1*) and carnitine palmitoyltransferase I (*CPT1A*; Figure 4C). In addition, genes associated with BA pathways, including cholesterol 7 alpha-hydroxylase (*CYP7A1*) and G protein–coupled bile acid receptor 1 (*GPBAR1*), as well as selected FXR-regulated genes, such as organic solute transporters (*SLC51A* and *SLC51B*), *NR0B2*, and *FETUB*, were downregulated (Figure 4C). These changes indicate impaired FXR activity in this in vitro model of experimental MASLD, which is consistent with the known role of FXR as a repressor of lipogenesis via inhibition of the liver X receptor (LXR)/SREBF1 pathway (36).

Exposure to Ibu and Pro also resulted in a significant induction of hepatic steatosis but with a lower magnitude and slower kinetics compared with FFAs (Figure 4A). Among lipogenic genes, both metabolites resulted in a comparable induction of *FASN* and *SLC13A5*, while only Pro demonstrated an increase in *PLIN1* expression (Figure 4, D and E). Transcripts involved in BA and FXR signaling remained largely unchanged, except for a marked reduction in *CYP7A1* observed only with Pro (Figure 4E). However, Pro showed a trend toward reduced expression of FXR-regulated genes, which is in contrast with Ibu (Figure 4, D and E). Furthermore, Pro resulted in a pronounced downregulation of *CPT1A* (Figure 4E), implicating suppressed fatty acid β-oxidation, alongside reductions in *G6PC1* and *CYP3A4*, key enzymes within glycolytic and cytochrome P450 metabolic pathways, respectively.

## Discussion

MASLD constitutes a spectrum of liver disease that is associated with substantial morbidity and mortality (37) throughout all stages, with a significant proportion of deaths resulting from cirrhosis, hepatocellular carcinoma, or extrahepatic cancer (38). While hepatic steatosis is benign in itself, it constitutes a key risk factor for the progression to MASH and fibrosis. As such, metabolomic signatures that allow early diagnosis of steatotic liver would be highly beneficial to flag patients for early intervention while the disease is still reversible. This is particularly true in lean individuals, where steatotic liver is typically underdiagnosed (39). In this study, we present data on circulating metabolites to determine metabolic signatures that are specific to lean and obese MASLD. Compared with previous investigations (6), our analysis was extended by measurement of BAs (40) and SCFAs (41), two classes of microbial metabolites that have been implicated in MASLD pathogenesis (34, 42). To the best of our knowledge, no reports on these metabolites and their differences and evidence of functional relevance related to MASLD and BMI are available.

Analysis of clinical and laboratory parameters showed that metabolic alterations (i.e., liver enzymes and lipid parameters) were present to a lesser degree in lean compared with obese MASLD. These findings are in accordance with a generally more favorable metabolic profile in lean MASLD (43), including milder features of the metabolic syndrome (44) and less insulin resistance. Notably, while most of these parameters were altered concomitantly in both MASLD etiologies, reduced IL-6 in participants with normal weight but not in participants with overweight was an exception and may imply differences related to inflammation and immune response (45).

The observed sex-related metabolomic profiles verified known metabolic pathway differences between men and women (29, 46), including unaltered serum SCFAs (47) and higher primary BAs in males (48). In addition, the positive correlation of branched-chain amino acids (Ile and Leu) and creatinine (49) in men verified the expected sex differences related to muscle mass, while higher SM levels in women (50, 51) were in accordance with a rapid increase in plasma sphingolipids with age in women (52). Together, these findings demonstrate a conserved sexual dimorphism within metabolic pathways, indicating that sex is associated with a discriminative metabolite signature in the MASLD cohort.

Regarding metabolic changes associated with MASLD, our results validated previous findings (i.e., reduced lyso-PC 17:0 and PC O-36:3 in lean MASLD accompanied by elevated glutamate levels compared with BMI-matched healthy controls) (6), while analysis in BMI subgroups uncovered additional changes in the modified amino acids acetylornithine, methionine sulfoxide, and sarcosine. Sarcosine is involved in hepatic *S*-adenosylmethionine homeostasis (53) as a methylation product of glycine mediated by glycine *N*-methyltransferase (GNMT) (54), and its deletion results in the development of steatotic liver fibrosis in mice (55). In line with a contribution of GNMT to phospholipid methylation, concomitant changes in the profiles of PC lipids, known to be utilized for methyl transfer reactions (56), point to a characteristic relationship between phospholipids and amino acid metabolism in obese MASLD. The observed higher level of sarcosine in women with obesity and MASLD, but not in men, is in accordance with previously described sex disparities in liver cancer susceptibility related to GNMT expression (57).

In contrast with obese MASLD, the SCFAs Ibu and Pro (but not acetate or butyrate) were among the most significantly enriched circulating metabolites in lean MASLD (Supplemental Table 3). Pro and Ibu are produced by the gut microbiota in the intestine via different pathways. Whereas Pro is generated through colonic digestion of dietary fibers, Ibu is a product of the fermentation of branched-chain amino acids, derived from undigested proteins (58). The observed differences in serum quantities may thus imply a higher production in lean MASLD possibly because of dysbiosis or dietary factors (59), consistent with a higher abundance of SCFA-producing bacteria in MASLD (60) and elevated concentrations of Pro and Ibu in fecal samples from patients with MASLD (61). In fact, the amount of fecal propionate was found to correlate with *Bacteroidetes* (62), a major bacterial phyla whose abundance was associated with nonobese MASLD (63). Another study showed that increased fiber intake ameliorated steatosis accompanied by diminished serum level of Pro, thereby linking diet to specific changes of microbial metabolites (64).

From a mechanistic point of view, several scenarios may underlie the observed changes of circulating microbial metabolites. Serum levels can be affected by differences in SCFA fluxes (65), such as increased metabolite efflux from the liver (66), or by a “selective” portal-systemic spillover (i.e., because of reduced hepatic uptake or higher intestinal production). Interestingly, downregulation of hepatic SLC16A1/MCT1 levels, the assumed substrate transporter for SCFAs, was observed in different liver pathology states, including alcoholic liver disease (67). However, to the best of our knowledge, no similar data for MASLD have been reported. To test the molecular consequences of an increased exposure of hepatocytes to SCFAs, we investigated the effects of Pro and Ibu on lipid accumulation and gene expression in human liver spheroids. These experiments demonstrated that both SCFAs resulted in increased steatosis, albeit to a lower extent than FFAs. Remarkably, even though phenotypic consequences appeared similar, the molecular impacts on gene expression patterns were different. FFAs induced the expression of genes involved in lipid metabolism and repressed FXR signaling, whereas Ibu shared the effects on lipid metabolism but notably did not inhibit FXR.

In contrast, Pro displayed distinct effects on lipogenic gene expression, particularly in its repression of *CPT1A*, suggesting reduced fatty acid oxidation, a common feature of steatosis (68). Additionally, the pronounced depletion of *G6PC1* in the presence of Pro points to reduced gluconeogenesis, aligning with the reported downregulation of the gene by Pro in HepG2 cells via the FFAR2/AMP-activated kinase (AMPK) pathway (69). The AMPK-dependent phosphorylation and subsequent inactivation of FXR (70) may further explain the Pro-induced inhibition of FXR signaling. Taken together, these findings suggest that the SCFAs Ibu and Pro exert specific and distinct effects on hepatic metabolism. While both induce lipogenic gene expression, even if the latter shows differences in the induction of individual genes, Pro additionally demonstrated impaired FXR activity. Overall, the pattern of genes regulated by Pro more closely resembles that induced by FFAs compared with the pattern observed with Ibu.

One limitation of the study is the sample size; however, the number of participants with MASLD and obesity comprised all participants fulfilling the inclusion criteria at the time of the first analysis. Hence, these phenotypes represent the rare and limiting manifestation of metabolic diseases in the background population. Whereas our investigations have provided better understanding of why lean and obese MASLD occur, the resulting signature may potentially be the basis for future biomarker development, which is beyond the scope of this work. In this regard, confirmation in independent cohorts is required to evaluate the generalizability of the observed significant findings. Another limitation is the lack of metagenomic data of the patients’ microbiome, as the study material was not prospectively collected. Moreover, histological assessments by liver biopsy were not conducted. The focus of the present study is on earlier stages of MASLD, and it is thus not possible to formally exclude patients with advanced fibrosis. As for not all participants a glucose tolerance test was carried out, diabetes assessment was done based on fasting glucose as a proxy (9). Hence, for patients lacking information from oral glucose tolerance test but displaying normal blood sugar levels, diabetes mellitus cannot be excluded. Moreover, expression profiling was limited to pathways relevant for steatotic liver and drug metabolism. Therefore, comprehensive profiling by RNA-Seq will allow us to extend these findings to global transcriptomic alterations as part of future investigations. Last, statistical analysis of the BA and SCFA data was carried out alongside metabolomics data previously acquired by the commercially available Biocrates p180 kit. Although the data for BAs and SCFAs were obtained using specific liquid chromatography–mass spectrometry (LC/MS) assays, the kit-based metabolite quantification partially relies on flow injection analysis coupled to mass spectrometry (FIA-MS). A limitation of FIA-MS, compared with LC/MS, is the inability to distinguish isobaric lipids (e.g., SM 33:2;3 versus SM 34:1;2 versus SM 35:0;1 or PC 33:1 versus PC O-34:1) in the mass spectrometer alone because of the lack of chromatographic separation. This limitation reduces confidence in the accuracy of the annotation of these lipid species provided by the commercial assay.

In summary, using comprehensive metabolic profiling, we demonstrate that circulating metabolites differ drastically between people with normal weight and obesity. Compared with conventional ultrasound for the diagnosis of MASLD, the use of circulating metabolites for a potential diagnosis may offer several advances, including (a) detection of early stages of disease, (b) provision of quantitative data that can be measured and tracked over time in a standardized manner, (c) pathophysiological insight into altered metabolic pathways, and (d) easy accessibility of blood samples with less physical contact and sometimes discomfort for patients undergoing ultrasound. The observed differences between lean and obese steatotic liver disease reinforce that lean MASLD is a molecularly distinct pathophenotype and provide strong evidence for a relationship between circulating SCFAs and alterations in hepatic lipid homeostasis. While the predictive value of individual microbial metabolites remains to be validated in independent clinical cohorts, functional validation in organotypic 3D liver cultures demonstrated that Pro and Ibu can induce lipid accumulation through specific modulation of lipogenesis, fatty acid metabolism, and BA signaling. These findings imply that various structurally diverse molecules beyond carbohydrates and FFAs can induce hepatic lipid accumulation. Our data thus suggest that microbial metabolites should be considered not only for minimally invasive diagnostics of MASLD but also to gain insights into their localized effects on hepatocyte function. Here, techniques such as MALDI-MSI (71) could provide valuable insights into the spatial metabolic changes of microbial metabolites in lean and obese MASLD, thereby opening up possibilities for a more detailed mechanistic exploration.

## Methods

### Sex as a biological variable.

Our study examined male and female participants. Sex differences are reported and were considered as a biological variable. We did not observe sex-specific differences in serum SCFA levels and therefore conducted our in vitro experiments using liver spheroids with donors of both sexes.

### Study cohort and metabolomics analysis.

A subset of 204 participants (self-identified as White and of European descent), recruited from the SAKKOPI study (6), were allocated to 4 groups: lean healthy (*n* = 61), lean MASLD (*n* = 49), obese healthy (*n* = 47), and obese MASLD (*n* = 47) (Table 1 and Figure 1). People with MASLD had unequivocal ultrasound evidence of steatotic liver whereas healthy people had normal ultrasound and biochemical liver tests. Specifically, the liver was considered normal if the echogenicity was homogenous and similar to or slightly higher than the echogenicity of the right renal parenchyma. The liver was considered steatotic if increased echogenicity was found in relation to the renal parenchyma. The severity of sonographic steatosis was not graded. The diagnosis of MASLD was therefore based on the findings on right upper quadrant ultrasound examination. Further details on definition of study groups, diagnosis, and endogenous metabolites associated with steatotic liver disease were described previously (6). Serum samples were randomized prior to quantitative BA and SCFA analysis using simple unrestricted randomization. BAs were quantified as described (40) with the following concentration ranges: 7.8–1,000 nM (GLCA, TUDCA, TDCA, and TLCA), 15.6–2,000 nM (GUDCA and TCA), 23.4–3,000 nM (LCA), 31.3–4,000 nM (CA, UDCA, DCA, and TCDCA), 46.9–6,000 nM (CDCA), 62.5–8,000 nM (GCA), 78.1–10,000 nM (GDCA), and 156.3–20,000 nM (GCDCA). Serum concentrations of acetate (Ac), Pro, butyrate (Bu), and Ibu were quantified by targeted LC-MS/MS as described (41). Serum was subjected to acetonitrile precipitation before derivatization. Briefly, 10 μL of serum was mixed with 10 μL of internal standard mixture. Then 100 μL of acetonitrile was added and mixed for protein precipitation. After centrifugation (19,000*g*), 50 μL of the supernatant was derivatized. An injection volume of 10 μL was used for LC-MS/MS analysis.

### Primary human hepatocyte spheroid culture.

Cryopreserved primary human hepatocytes (PHHs) were obtained from BioIVT (Westbury) with prior consent from the donors. Three donors were assessed: donor 1 (male, 25 years, BMI: 32.2), donor 2 (female, 27 years, BMI: 28.2), and donor 3 (female, 57 years, BMI: 21.3). The PHHs were seeded as previously described (72). Briefly, 1,500 viable cells were seeded into each well of a 96-well ultralow-attachment plate (Corning) in 100 μL William’s E medium supplemented with 11 mM glucose, 10 mg/L insulin, 100 nM dexamethasone, 5.5 mg/L transferrin, 6.7 μg/L selenite, 2 mM l-glutamine, 1 U/mL penicillin, 0.1 mg/mL streptomycin, and 10% fetal bovine serum (FBS). Spontaneous aggregation occurred on day 7 after seeding and FBS was phased out. The PHHs were then maintained in the same media, treated with 0.8% albumin-conjugated FFAs (comprising 320 μM palmitic acid and 320 μM oleic acid), or treated with various doses of the metabolites as indicated in the main text. After 1 and 2 weeks, triglyceride levels in each spheroid were quantified by AdipoRed adipogenesis assay (Lonza) with a fluorescence readout (excitation/emission = 485 nm/572 nm).

### Gene expression analysis in RNA isolates from liver spheroids.

PHH spheroids were pooled (24–32 per replicate). RNA was extracted with a Quick-RNA MiniPrep (Zymo Research, catalog R1055) according to the manufacturer’s instruction. cDNA was then synthesized with SuperScript III reverse transcriptase (Thermo Fisher Scientific, catalog 18080093) and kept at –80°C before use. Relative quantification of gene expression (2^–*ΔΔ*Ct^ method) was performed by TaqMan RT-qPCR (Thermo Fisher Scientific) using the Biomark HD system and 48.48 Gene Expression Integrated Fluidic Circuits (Standard BioTools), as described previously (73), using 20 preamplification cycles of cDNA samples, which originally corresponded to 1.6 ng of reverse-transcribed RNA. Analysis was run in technical triplicates. Genes, associated with glucose and lipid metabolism, as well as regulated in BA- and FXR-dependent signaling and encoding selected cytochrome P450 genes and SCFA receptors, were selected for analysis (Supplemental Figure 1). Supplemental Table 1 shows the commercial TaqMan gene expression assays used, consisting of validated predesigned primer/probe sets (Thermo Fisher Scientific). Respective gene expression levels were normalized to the corresponding expression level of *YWHAZ*, which demonstrated stability in experimental MASLD (74), thereby generating *ΔCt* values. ΔΔ*Ct* values for each gene were calculated by subtracting the average Δ*Ct* of the control group from the Δ*Ct* values of individual samples of control and treatment groups (FFA, Ibu, and Pro). These values were transformed using the 2−ΔΔ*Ct* formula and log_2_-transformed for statistical analysis.

### Statistics.

Prior to statistical analysis BA species with values below the limit of quantification (LOQ) in more than 80% of analyzed study samples were removed from the data set (i.e., TLCA and TUDCA) and thus not considered. Concentration values below LOQ of remaining species were used for further statistical analysis (75). BAs were grouped as total BAs (sum of all species), unconjugated BAs (sum of unconjugated species), glycine-conjugated BAs (sum of glycine-conjugated species), taurine-conjugated BAs (sum of taurine-conjugated species), total primary (sum of conjugated and unconjugated CA and CDCA), total secondary (sum of conjugated and unconjugated DCA, UDCA, and LCA), total conjugated primary (sum of conjugated CA and CDCA), total unconjugated primary (sum of unconjugated CA and CDCA), total conjugated secondary (sum of conjugated DCA, UDCA, and LCA), total unconjugated secondary (sum of unconjugated DCA, UDCA, and LCA), ratio CA to CDCA, and the ratio total CA (sum of conjugated and unconjugated CA) to total CDCA (sum of conjugated and unconjugated CDCA). SCFAs were grouped as total (sum of Ac, Pro, Bu, and Ibu) or considered as single species. BA and SCFA data were merged with laboratory and metabolomics data that were acquired previously (6), thereby resulting in a total of *n* = 179 metabolic traits. Prior to statistical analyses, metabolite data were transformed by generalized log_2_ (76). Metabolic signatures that differed significantly between men and women as well as between MASLD and healthy groups were assessed by linear model analysis. Classification of steatosis versus healthy in patients with normal weight and obesity was performed applying LASSO regression with 100 times repeated 10-fold nested cross-validation (77). For each of the 100 repeats, area under receiver operating characteristics curve was calculated and used to compute median, minimum, and maximum AUC values. For correction of multiple testing, the Benjamini-Hochberg (78) procedure was applied. All statistical tests were 2 sided, and the significance level was defined as 0.05. Kruskal-Wallis tests and drawing of volcano and box plots were prepared with GraphPad Prism (Version 9.3.1). The Venn diagram was created based on significantly altered metabolites (Supplemental Tables 2–5) with Venny 2.1 (https://bioinfogp.cnb.csic.es/tools/venny/). All other statistical analysis were performed in the R studio statistics software (79) version 4.0.5 with the additional packages limma_3.46.0 (80, 81), glmnet_4.1-6 (82), ROCR_1.0-11 (83), and cvAUC_1.1.4 (84). Group differences in gene expression data were assessed with 1-way ANOVA, followed by Dunnett’s multiple-comparison test in GraphPad Prism to compare each treatment group against the control group. Genes of interest were defined as those exhibiting significant changes (adjusted *P* value < 0.05 and log_2_ fold-change > 0.5) in at least 1 group comparison for a single donor with a consistent trend observed in the second donor.

### Study approval.

The study was approved by the local ethics committee in Salzburg, Austria, and written informed consent was obtained from all participants.

### Data availability.

Supporting data values of figures and Table 1 are available as a supplemental Supporting Data Values file. Metabolomics data can be accessed via FigShare (https://figshare.com/) at https://doi.org/10.6084/m9.figshare.27890241.v1

## Author contributions

MH and SW conducted data analysis, performed investigations, and drafted and wrote the manuscript. AMK, GL, and OB were involved in data analysis, investigation, and the revision of the manuscript for important intellectual content. JT, AF, SKE, TKF, CD, and BP provided resources, participated in patient recruitment, contributed to data acquisition, and revised the manuscript for important intellectual content. VML, EA, and MS contributed to the study concept and design, supervised the study, and outlined and revised the manuscript. All authors have read and approved the final manuscript.

## Supplementary Material

Supplemental data

ICMJE disclosure forms

Supplemental tables 1-6

Supporting data values

## Figures and Tables

**Table 1:**
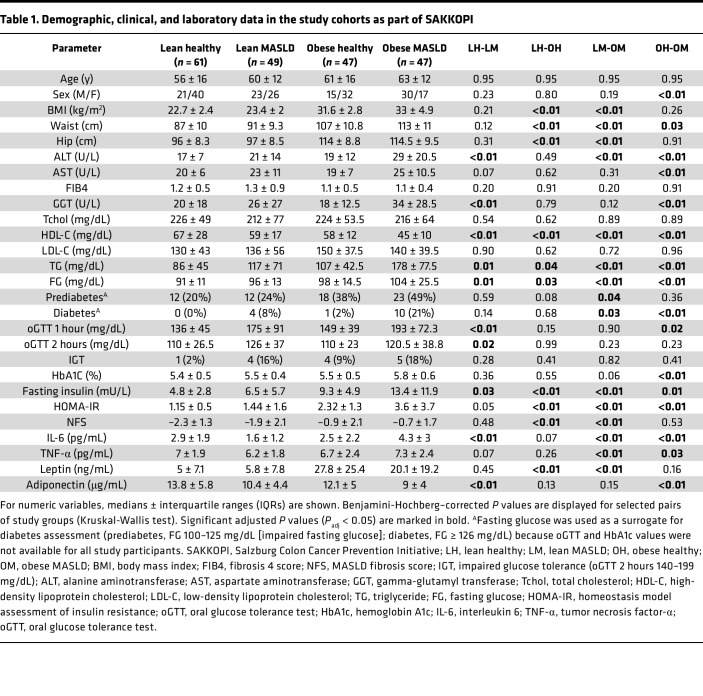
Demographic, clinical, and laboratory data in the study cohorts as part of SAKKOPI

## Version 1 03/18/2025

In-Press Preview

## Version 2 04/22/2025

Electronic publication

## Version 3 04/24/2025

The Methods and Affiliations were updated
