# Supplementary material for: Circulating metabolite signatures indicate differential gut-liver crosstalk in lean and obese MASLD
Source: JCI Insight. 2025 Mar 18;10(8):e180943. doi: 10.1172/jci.insight.180943 (PMC12016937; doi:10.1172/jci.insight.180943)
Supplement: Supplemental data [file jciinsight-10-180943-s006.pdf]

Our study is not a clinical trial involving patient recruitment, but uses data and biomaterials from a previous study (Feldman *et al.*, 2017, PMID: 27527746). Subjects were recruited from ~2,500 participants of the underlying non-interventional colon carcinoma screening study (Salzburg Colon Cancer Prevention Initiative). The study design and details of the clinical and biochemical work-up of the included subjects have been reported previously (Stadlmayr *et al.*, 2011, PMID: 21414047).

This publication includes:

Subjects
The study cohort consisted of 1382 consecutive Caucasians (702 males, 40–76 years; 680 females, 31–88 years) who underwent colonoscopy for CRC screening according to national screening recommendations at a single centre between June 2007 and December 2009. The study was approved by the local ethics committee, and informed consent was obtained from all participants.

Study concept and execution
Study participants were examined on two consecutive days, and venous blood was collected and analysed following an overnight fast. In series, a medical history was taken, and dietary practices were assessed using a validated food-frequency questionnaire. A physical examination and right upper quadrant US examination were performed. On the second day, all subjects underwent complete colonoscopy.

Laboratory assessment
Full blood counts were obtained in all subjects by standard laboratory methods. Erythrocyte sedimentation rate was measured in citrated plasma. Blood was centrifuged, and plasma was analysed for aspartate aminotranspeptidase and alanine aminotranspeptidase activities and levels of triglycerides, total cholesterol, high-density and low-density lipoprotein cholesterol, glucose, insulin and C-reactive protein (CRP). Glycated haemoglobin A1c was measured by HPLC using an Adamts H-8160 analyzer (Menarini, Florence, Italy). The homeostasis model assessment-insulin resistance (HOMA-IR; fasting insulin [μU L^{-1}] × fasting glucose [mmol dL^{-1}]/22.5) was used to assess IR.

US examination
Right upper quadrant US examination was performed using an ATL HDI 5000 machine (Phillips Medical Systems, Vienna, Austria). The examinations were carried out by one of the five physicians with 5–21 years of experience. The liver was considered ‘normal’ if the echogenicity was homogenous and similar to or slightly higher than the echogenicity of the renal parenchyma. The liver was considered ‘fatty liver’ if areas of significant increased echogenicity in relation to the renal parenchyma were found. The severity of sonographic steatosis was not graded [19]. The diagnosis of NAFLD was based on specific findings on right upper quadrant US examination (as noted above) and after exclusion of viral, autoimmune and hereditary (Wilson’s disease, hereditary haemochromatosis, alpha-1 antitrypsin deficiency) liver disease and excess alcohol consumption (determined with a questionnaire; cut-off >20 g day^{-1}, a limit considered to be below the traditional level for alcohol-induced liver disease [20]).

A total of 632 patients (45.7%) had evidence of NAFLD on US examination. In patients with elevated liver enzymes and/or a positive US result suggestive of hepatic steatosis, serum was analysed for the evidence of unrecognized hepatitis C virus infection, hepatitis B virus infection, autoimmune hepatitis (AIH), Wilson’s disease or alpha-1 antitrypsin deficiency.

Subjects with increased serum ferritin or transferrin saturation underwent *HFE* genotyping to exclude hereditary haemochromatosis. Ninety-nine (14.1%) male subjects were excluded because of incomplete colonoscopies ($n = 10$), a history of recent colorectal polypectomy ($n = 1$), asymptomatic ulcerative colitis ($n = 3$), other extraintestinal malignancies ($n = 3$), viral hepatitis ($n = 10$), AIH ($n = 1$), homozygous hereditary haemochromatosis ($n = 3$), a history of excess alcohol consumption (>20 g day^{-1}, $n = 34$) or refusal to participate in the study ($n = 34$). Seventy-two (10.6%) women were excluded because of incomplete colonoscopies ($n = 18$), a history of recent colorectal polypectomy ($n = 2$), asymptomatic ulcerative colitis ($n = 1$), asymptomatic Crohn's disease ($n = 2$), other extraintestinal malignancies ($n = 2$), viral hepatitis ($n = 17$), AIH ($n = 1$), a history of excess alcohol consumption (>20 g day^{-1}, $n = 10$) or refusal to participate ($n = 19$). Thus, data from 603 males (85.9%) and 608 females (89.4%) were included in the final analysis.

Metabolic syndrome was evaluated as defined by the National Cholesterol Education Program Adult Treatment Panel [21]. Blood pressure was determined twice by a nurse after a 5-min rest in a sitting position, and the average was taken as the measurement of blood pressure. Waist circumference was measured at the highest point of the iliac crest with subjects standing in an upright position. The MetS was diagnosed when three or more of the following criteria were met: fasting blood glucose level ≥6.1 mmol L^{-1}, waist circumference >102 cm in males and >88 cm in females, blood pressure ≥130/85 mmHg or current antihypertensive treatment, plasma triglycerides ≥1.7 mmol L^{-1} and plasma HDL <1.0 mmol L^{-1} in males and <1.3 mmol L^{-1} in females. Body mass index (BMI) was calculated as weight/height squared (kg m^{-2}).

Colonoscopy

The laxative Klean–Prep® (containing macrogol 59.0 g, sodium sulphate 5.68 g, sodium bicarbonate 1.68 g, NaCl 1.46 g and potassium chloride 0.74 g; Norgine, Marburg, Germany) was used for bowel preparation before colonoscopy. Colonoscopic findings were classified as tubular adenoma, advanced adenoma, i.e. villous or tubulovillous features, size ≥1 cm or high-grade dysplasia [22, 23] or carcinoma after a combined analysis of macroscopic and histological results. Lesions were classified by location (i.e. proximal colon including caecum, ascending colon and transverse colon, distal colon ranging from the splenic flexure to the sigmoid and rectum alone) [24].

Statistical analysis

SigmaStat 3.1 or STATA 0.8 software packages (Erkrath, Germany) were used for all analyses. Data are presented as mean ± SD, unless otherwise indicated. Student's *t*-test was used for comparison of continuous variables or Mann–Whitney *U*-test in the case of non-Gaussian distribution of variables. The chi-square or McNemar's test was used to calculate rates and proportions as appropriate. Multivariate regression analysis was used to determine variables independently associated with the number of polyps. Logistic regression analysis was used to determine variables predicting the presence of adenoma. As this was a cross-sectional study, the dependent variable in the logistic regression equation is the log odds of having adenoma at the time of examination. Throughout, a two-tailed *P*-value <0.05 was considered statistically significant.

Please note that an official study protocol was not required by the *Journal of Internal Medicine* (JIM), and therefore, it was not made available for download.

Supplemental Figures

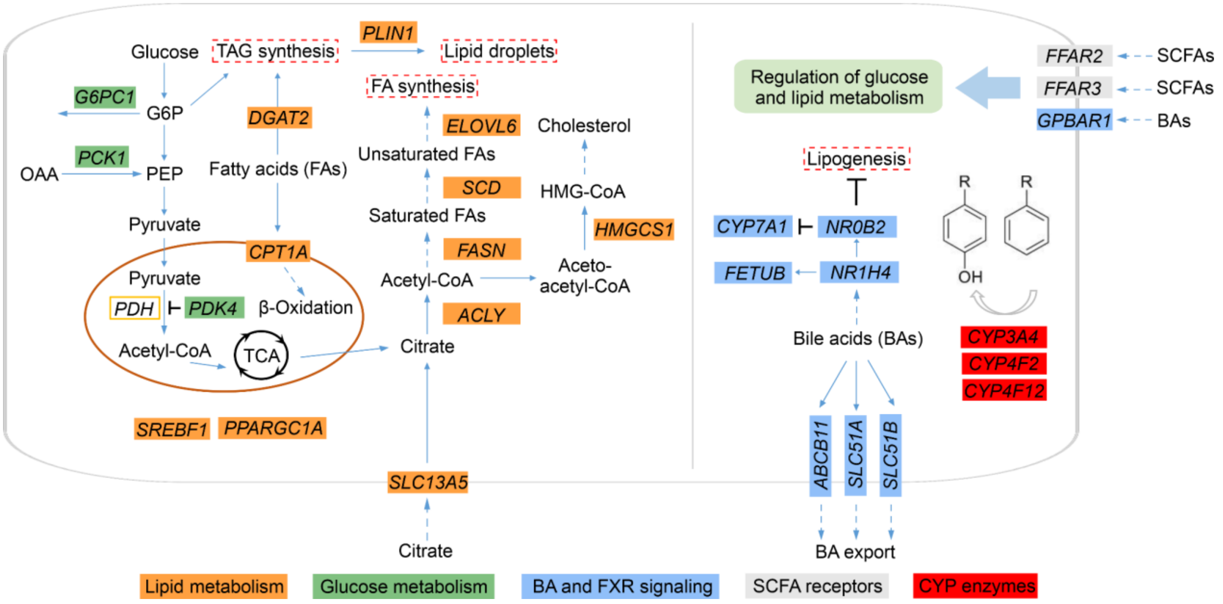

Supplemental Figure 1: **Schematic representation of the transcripts related to the indicated pathways that were assessed for gene expression analysis**. Abbreviations: OAA, oxaloacetate; G6P, glucose-6-phosphate; PEP, phosphoenolpyruvate; HMG-CoA, hydroxymethylglutaryl-CoA.

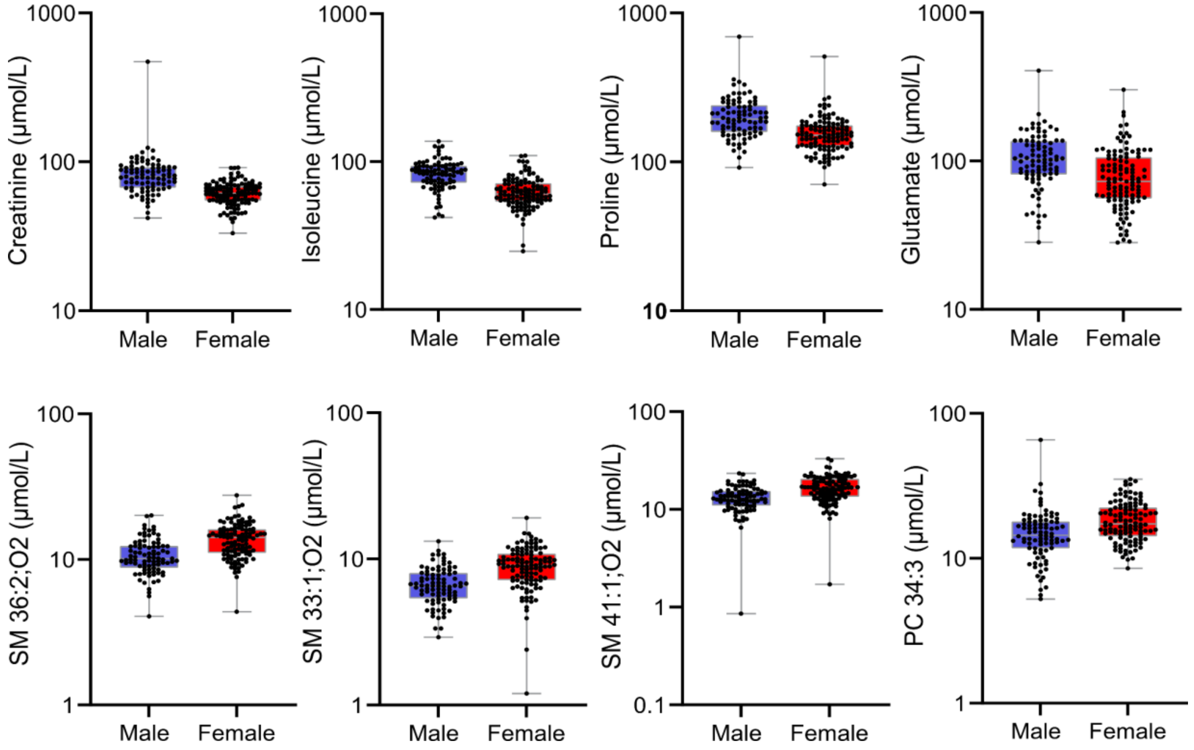

Supplemental Figure 2: **Box-plots of circulating metabolites that are relevantly (log2 fold change >0.3125) and significantly (adjusted p-value <0.05) different between males and females**. Box-plots of metabolites with highest absolute log2 fold changes are shown in Figure 2C. The upper/lower borders of a box are defined by the 1st/3rd quartile while the line within a box represents the median. Whiskers extend to the highest or lowest values.

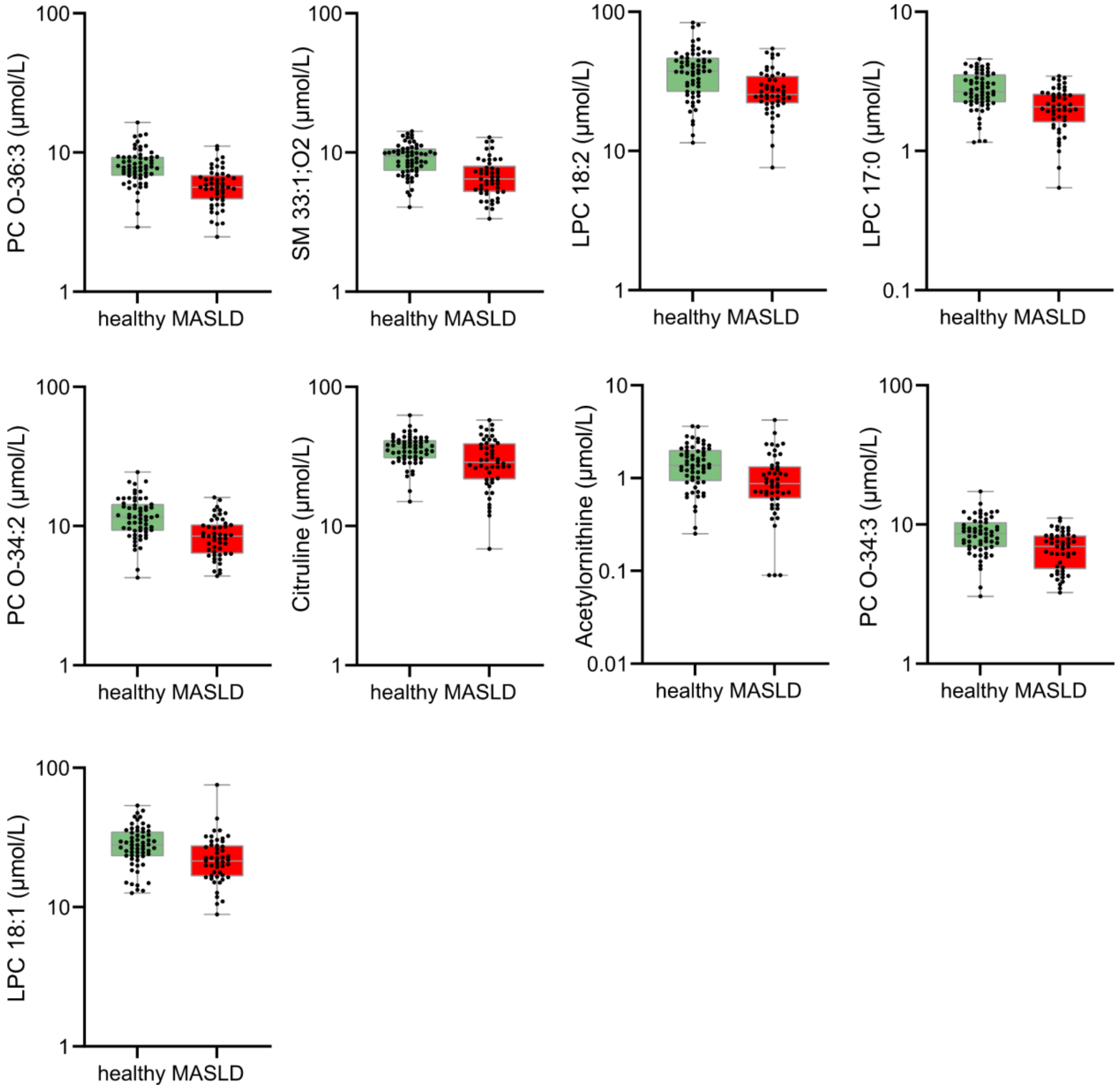

Supplemental Figure 3: **Box-plots of circulating metabolites that are relevantly (log2 fold change >0.3125) and significantly (adjusted p-value <0.05) reduced in lean patients with MASLD compared to lean healthy patients** (see corresponding volcano plot in Figure 2D). The upper/lower borders of a box are defined by the 1st/3rd quartile while the line within a box represents the median. Whiskers extend to the highest or lowest values.

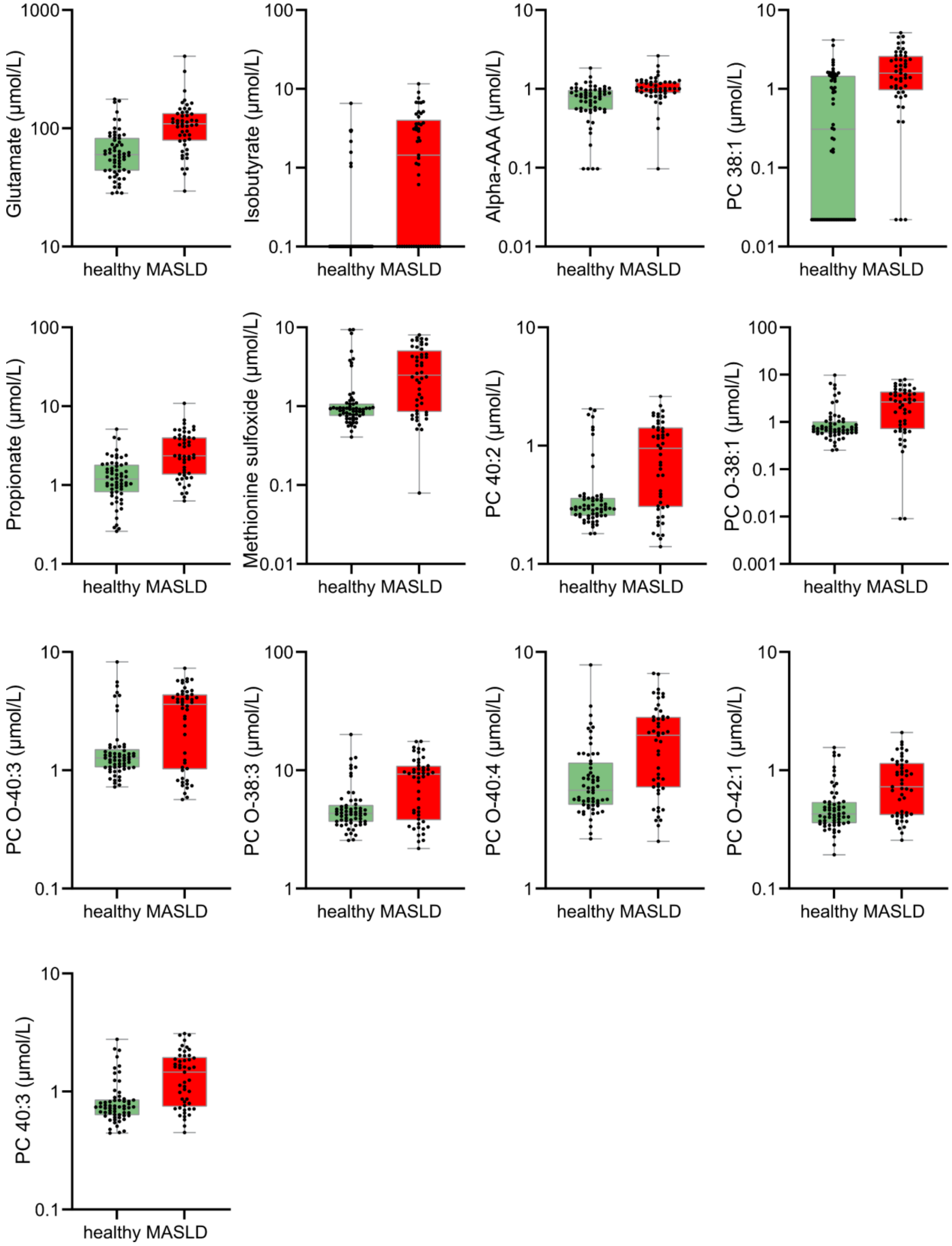

Supplemental Figure 4: **Box-plots of circulating metabolites that are relevantly (log2 fold change >0.3125) and significantly (adjusted p-value <0.05) elevated in lean patients with MASLD compared to lean healthy patients** (see corresponding volcano plot in Figure 2D). The upper/lower borders of a box are defined by the 1st/3rd quartile while the line within a box represents the median. Whiskers extend to the highest or lowest values.

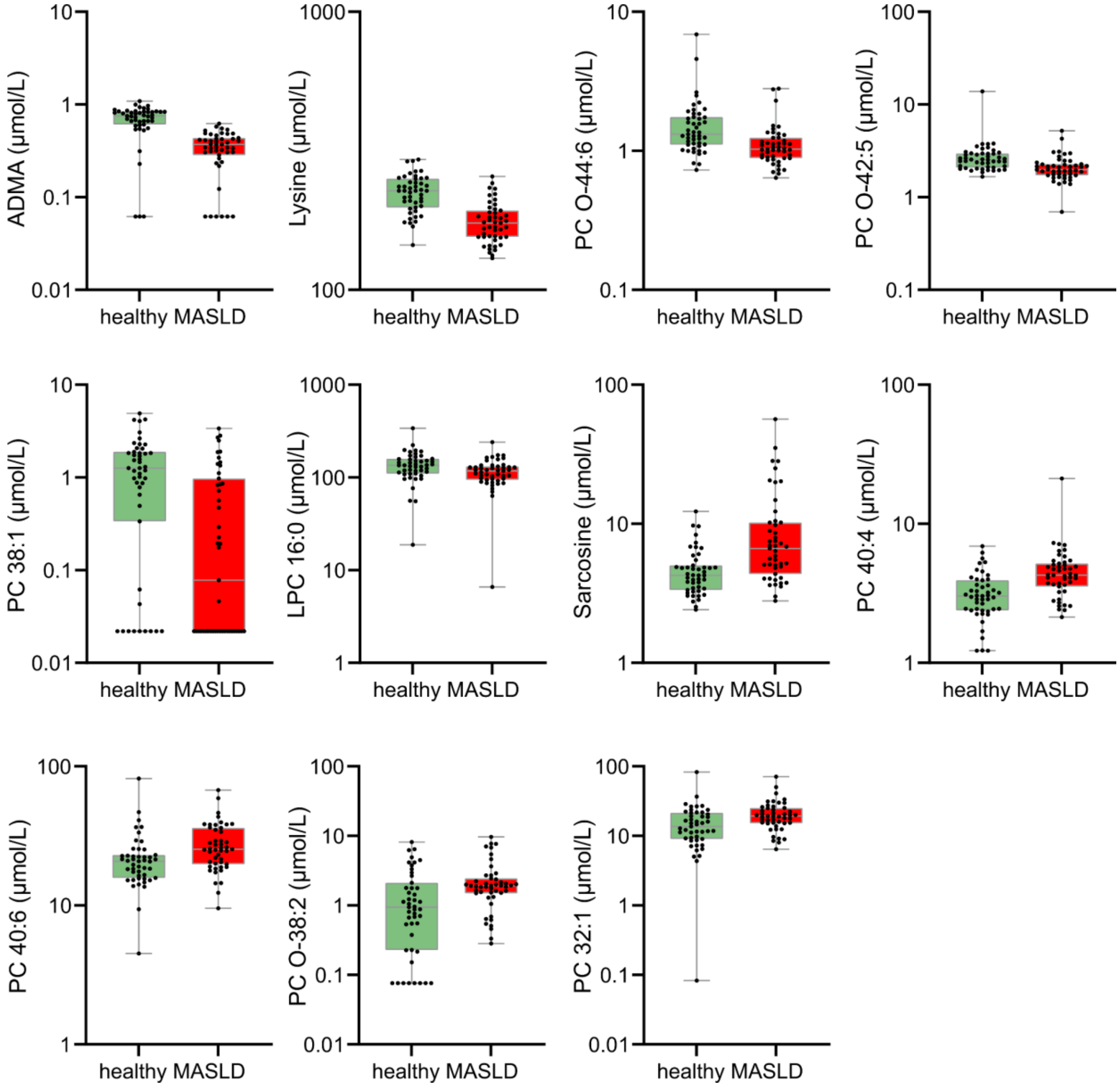

Supplemental Figure 5: **Box-plots of circulating metabolites that are relevantly (log2 fold change >0.3125) and significantly (adjusted p-value <0.05) altered in patients with obesity and MASLD compared to healthy patients with obesity** (see corresponding volcano plot in Figure 2E). The upper/lower borders of a box are defined by the 1st/3rd quartile while the line within a box represents the median. Whiskers extend to the highest or lowest values.

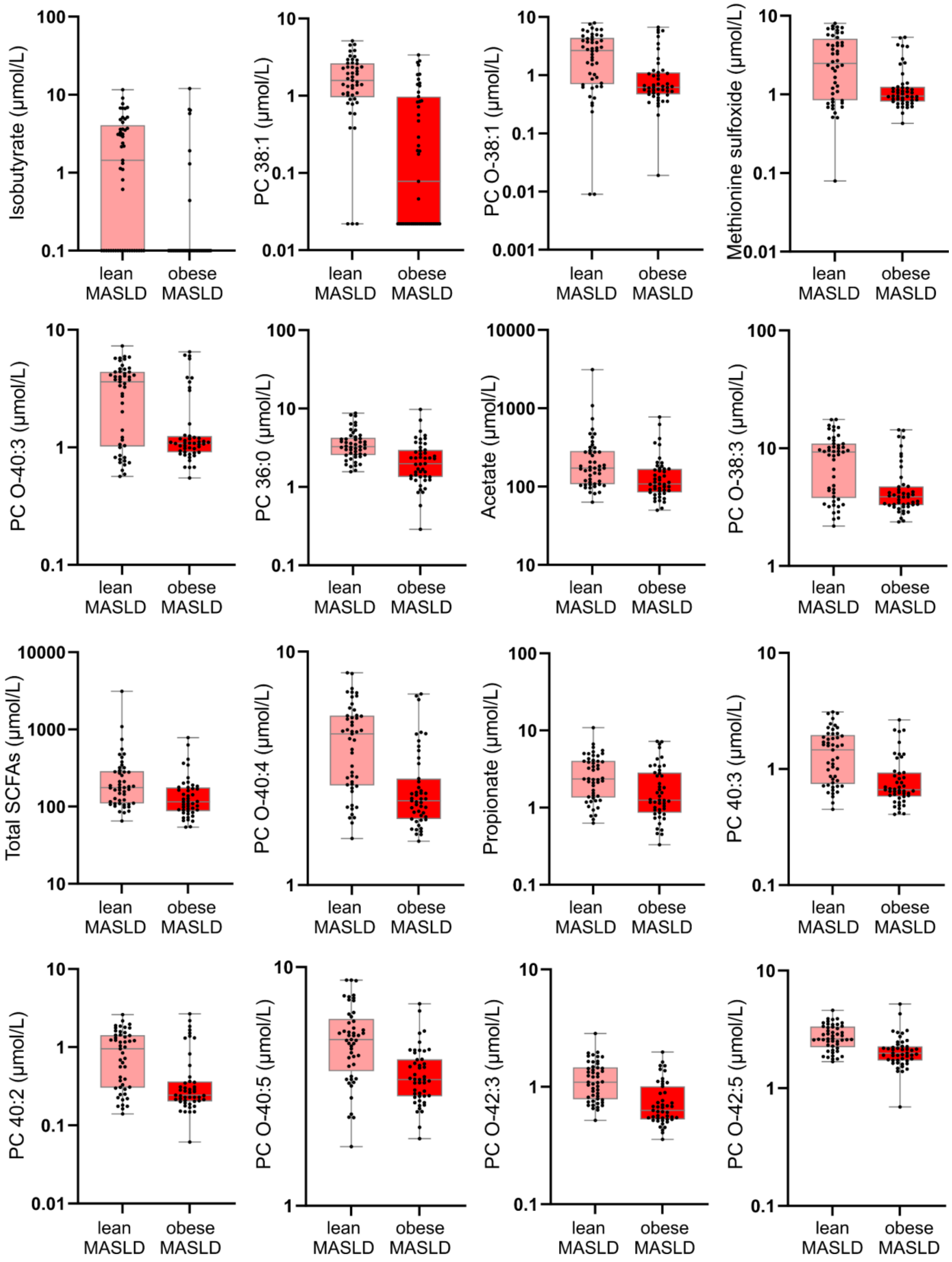

Supplemental Figure 6: **Box-plots of circulating metabolites that are relevantly (log2 fold change >0.3125) and significantly (adjusted p-value <0.05) altered in patients with obesity and MASLD compared to lean patients with MASLD** (see corresponding volcano plot in Figure 2F). The upper/lower borders of a box are defined by the 1st/3rd quartile while the line within a box represents the median. Whiskers extend to the highest or lowest values.

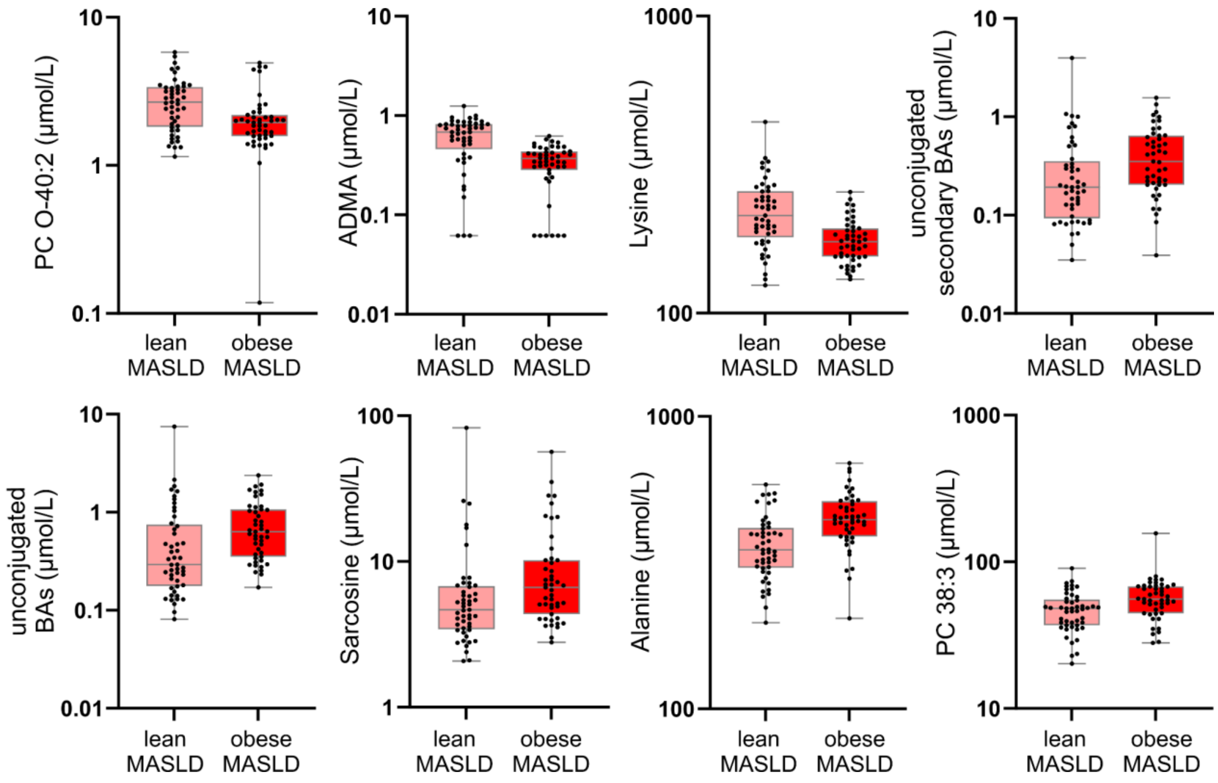

Supplemental Figure 7: **Box-plots of circulating metabolites that are relevantly (log2 fold change >0.3125) and significantly (adjusted p-value <0.05) altered in patients with obesity and MASLD compared to lean patients with MASLD** (see corresponding volcano plot in Figure 2F). The upper/lower borders of a box are defined by the 1st/3rd quartile while the line within a box represents the median. Whiskers extend to the highest or lowest values.

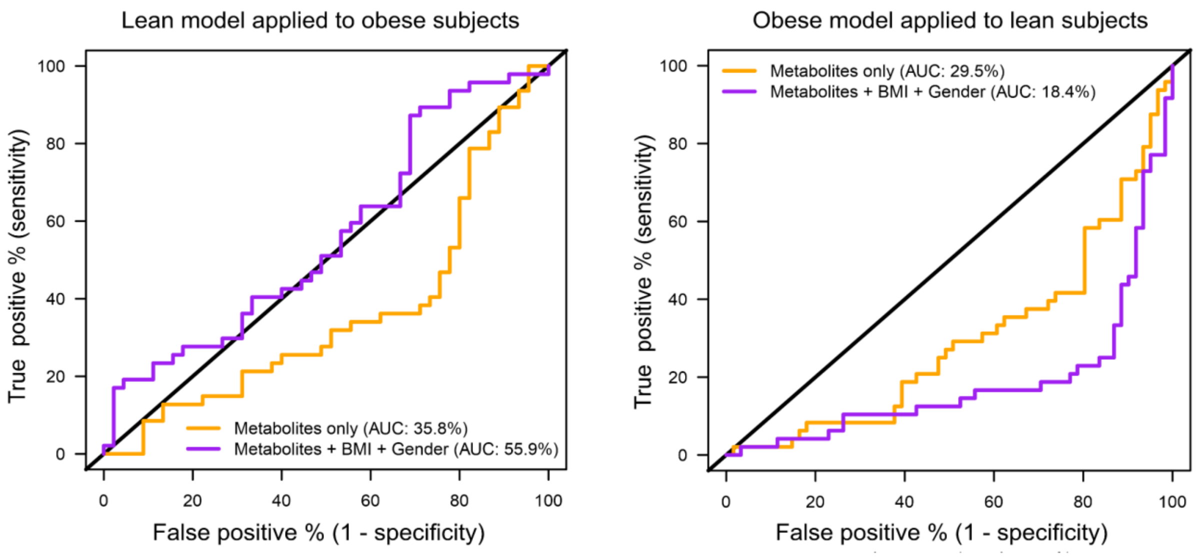

	Lean model applied to obese subjects	Obese model applied to lean subjects
Met only	35.8 [35.1,36.9]	29.5 [28.6,30.3]
Met, BMI & gender	55.9 [52.8,69.7]	18.4 [18.2,19.1]

Supplemental Figure 8: **MASLD** ***vs.*** **healthy prediction in lean and obese subgroups** considering metabolites only (yellow curve) or metabolites + gender + BMI (magenta curve) when applying the metabolite-based models (with or without BMI and gender covariates) established on the lean subgroups to the obese subgroups or vice versa. Values in the table indicate median AUCs [min./max.].
